# A Novel PVDF Ultrafiltration Membrane Modified by C_60_(OH)_n_-Ag

**DOI:** 10.3390/polym16233359

**Published:** 2024-11-29

**Authors:** Jie Zhang, Wenjun Zhao, Chengyang Shi, Liman Zhao, Yudi Chu, Yanan Ren, Qun Wang, Yanxia Chi, Shujing Zhou

**Affiliations:** Heilongjiang Provincial Key Laboratory of New Drug Development and Pharmacotoxicological Evaluation, Jiamusi University, Jiamusi 154007, China; zjie612@163.com (J.Z.); 15935446412@139.com (W.Z.); shichengyang@163.com (C.S.); 15645415045@163.com (L.Z.); 15124694551@163.com (Y.C.); renyanan2623@163.com (Y.R.); wq13614699333@163.com (Q.W.); yanxiachi123@163.com (Y.C.)

**Keywords:** ultrafiltration membrane, PVDF, C_60_, silver, organic pollutant

## Abstract

Ultrafiltration membranes in the fields of water treatment and biomedicine should have high permeability as well as antibacterial and antifouling capabilities. In this study, based on the hydrophilicity of fullerol (C_60_(OH)_n_) and the bacteriostatic properties of silver (Ag), a fullerol–silver (C_60_(OH)_n_-Ag) complex was prepared as a multifunctional additive. A polyvinylidene fluoride (PVDF)-composited C_60_(OH)_n_-Ag ultrafiltration membrane (C_60_(OH)_n_-Ag/PVDF) was prepared by immersion precipitation phase transformation. Addition of the C_60_(OH)_n_-Ag complex improved the permeability and retention of the traditional PVDF membrane. Compared with the traditional PVDF membrane, the surface water contact angle of the modified PVDF and C_60_(OH)_n_-Ag ultrafiltration membrane was reduced from 75.05° to 34.50°, its pure water flux increased from 224.11 L·m^−2^·h^−1^ to 804.05 L·m^−2^·h^−1^, the retention rate on bovine serum protein was increased from 75.00% to 96.44% and the flux recovery rate increased from 64.91% to 79.08%. The C_60_(OH)_n_-Ag/PVDF ultrafiltration membrane had good inhibitory effects on *Escherichia coli* and *Staphylococcus aureus*, while the PVDF ultrafiltration membrane had no obvious inhibitory effects.

## 1. Introduction

Ultrafiltration technology is regarded as a green wastewater recycling treatment strategy because of advantages such as high retention rate, low energy consumption and low secondary pollution [1,2]. PVDF ultrafiltration membranes play an important role in the ultrafiltration of products due to their flexibility, chemical stability and low cost [3,4]. However, due to the strong hydrophobicity of PVDF ultrafiltration membranes, various organic compounds and pathogenic microorganisms easily adhere to the membrane surface or are trapped in the membrane pores; as a result, it is easy to cause membrane pollution [5,6]. PVDF membrane pollution can lead to increased energy operating costs, large chemical consumption, and shortened membrane life. Therefore, it is of great significance to improve the hydrophilicity of PVDF membranes.

Blending is one of the simplest and most effective methods of improving membrane properties. In recent years, carbon nanomaterials, such as graphene oxide [6,7,8,9] and carbon nanotubes [10,11], have been widely used as blending additives in the modification of PVDF ultrafiltration membranes, improving their pollution resistance, hydrophilicity, mechanical stability and thermal stability. It has been reported that PVDF ultrafiltration membranes modified by doping with a GO/TiO_2_ nanocomplex can double the water flux and maintain a high BSA retention rate, and the collaborative coupling reaction based on GO and TiO_2_ can give photocatalytic anti-fouling functions to PVDF ultrafiltration membranes [12]. Fullerene (C_60_), an engineering carbon nanomaterial, has excellent properties, such as anti-oxidation, antibacterial and chemical corrosion resistance, due to its unique structure [13,14]. However, the application of C_60_ in the modification of ultrafiltration membranes is rarely reported; the difficulty of doping is that C_60_ has poor water solubility, low solubility in organic solvents and strong hydrophobicity. Fullerol (C_60_(OH)_n_), as a derivative of C_60_, not only retains the performance advantages of C_60_ but also has hydrophilic properties [15]. In addition, the properties of C_60_(OH)_n_, such as antioxidant, antibacterial and cytoprotective effects, have been further enhanced compared to C_60_ [16]. Using C_60_(OH)_n_ as a blend additive can enhance the hydrophilicity of PVDF ultrafiltration membranes and give PVDF ultrafiltration membranes good antibacterial performance.

In this study, the C_60_(OH)_n_-Ag complex was proposed as a multifunctional additive to improve the hydrophilicity and fouling resistance of PVDF ultrafiltration membranes. The relationship between the structure and properties of the obtained C_60_(OH)_n_-Ag/PVDF ultrafiltration membrane was systematically studied in order to obtain a modified membrane with good hydrophilic properties and excellent antibacterial effects.

## 2. Experiment

### 2.1. Materials

Polyvinylidene fluoride (PVDF) in powder form was obtained from Shanghai Aofu Chemical Technology Co., Ltd. (Shanghai, China); the solvents *N*,*N*-2-methylformamide (DMF), polyvinylpyrrolidone (PVP, K30), hydrogen peroxide (30%), silver nitrate and hydrochloric acid were purchased from Tianjin Kemio Chemical Reagent Co., Ltd. (Tianjin, China); fullerene (C_60_, 99.9%) was purchased from Suzhou Carbonfeng Graphene Technology Co., Ltd. (Suzhou, China); xylene, N-hexane and sodium citrate were purchased from Tianjin Kaitong Chemical Reagent Co., Ltd. (Tianjin, China); tetrabutyl ammonium hydroxide was purchased from Chengdu Aikeda Chemical Reagent Co., Ltd. (Chengdu, China); bovine serum albumin (BSA, M_w_ = 67,000), egg white protein (OVA M_w_ = 45,000), pepsin (PS M_w_ = 35,000), trypsin (TPS M_w_ = 25,000) were obtained from Shanghai Bio Life Science and Technology Co., Ltd. (Shanghai, China). The deionized water used in all experiments was supplied by a Millipore Elix water purification system(Shanghai ZHIYAN Scientific Instrument Co. Ltd, Shanghai, China). The reagents used in this experiment were all analytical grade. 

### 2.2. Preparation and Characterization of the C_60_(OH)_n_-Ag Complex

C_60_(OH)_n_ was prepared according to the typical method [17]. After the prepared C_60_(OH)_n_ (20 mg) was placed in deionized water (20 mL) and uniformly dispersed under ultrasound for 60 min, a C_60_(OH)_n_ aqueous solution (1 mg·mL^−1^) was obtained. Silver nitrate (8.5 mg) was added to the above solution, the whole system was stirred for 10 min and then trisodium citrate (14.8 mg) was added. After stirring in a water bath at 60 °C for 2 h, the above solid–liquid mixture was centrifuged at 10,000 r/min for 5 min, and the C_60_(OH)_n_-Ag complex was obtained by vacuum drying at 50 °C for 24 h.

The obtained C_60_(OH)_n_-Ag complex was characterized using a Fourier transform infrared attenuation full reflection infrared spectrometer (ATR-FTIR), X-ray diffraction (XRD), a nanoparticle size analyzer, and an X-ray energy spectrometer (EDS).

### 2.3. Preparation of the Ultrafiltration Membrane

C_60_(OH)_n_ or C_60_(OH)_n_-Ag was ultrasonically dispersed in dimethylformamide (DMF), and then polyvinylidene fluoride (PVDF) and polyvinylpyrrolidone (PVP) were added. The system was then heated and stirred until a transparent and uniform casting film liquid was formed, and the static defoaming was performed. The casting film liquid was cast on the glass plate; the scraping film process was performed by using a film scraper of 200 μm. The glass plate containing the casting film liquid was immersed in the water solidification bath. The shed membrane was soaked in distilled water for 24 h. The PVDF ultrafiltration membrane, C_60_(OH)_n_/PVDF ultrafiltration membrane and C_60_(OH)_n_-Ag/PVDF ultrafiltration membrane were, respectively, obtained.

### 2.4. Performance Analysis of the Ultrafiltration Membrane

The chemical composition and structural information of materials were obtained using a UV spectrophotometer (T6, Guangdong Solid Ultrasound Co, Ltd., Meizhou, China), a Fourier transform infrared attenuation full reflection infrared spectrometer (ATR-FTIR; Thermo iS50, Shanghai Lairui Scientific Instrument Co., Ltd., Shanghai, China), and an X-ray energy spectrometer (EDS, JSM-6460LV, Japan Electronics Co., Ltd., Tokyo, Japan). The surface morphologies of the samples were analyzed using SEM (JSM-6460LV, Japan Electronics Co., Ltd., Tokyo, Japan) and atomic force microscopy (AFM, Dimension ICON, Brooke Technology Ltd., Hanover, Germany). The surface potential changes in the sample were recorded on a solid surface Zeta potential tester (SURPASS3, Andonpa Technology Co., Ltd., Hong Kong, China).

#### 2.4.1. Physical Performance Analysis

Contact angles were measured using a contact angle tester (JCY-1, Shanghai Fangrui Instrument Factory, Shanghai, China). The tensile strength was tested using a universal material testing machine (Jinan Dongchen Test Instrument Factory, Jinan, China).

The gravimetric method and the Guerout–Elford–Ferry equation were used to calculate the pore size (ε) [18] and average pore size [19] of the prepared membrane, respectively, as shown in Formulas (1) and (2).

(1) $$\varepsilon \left(\%\right)=\frac{W_{w}-W_{d}}{\rho_{w}\cdot A\cdot T}\times 100\%$$

where ε is the porosity (%); W_w_ is the wet film weight (kg); W_d_ is the dry film weight (kg); ρ_w_ is the density of water (998 kg·m^−3)^; A is the effective area of the membrane (0.1 m^2^) and T is the thickness of the membrane (m).

(2) $$r=\sqrt{\frac{(2.9-1.75\varepsilon )\times 8\eta \mathrm{TQ}}{\varepsilon \times A\times \Delta P}}$$

where η is the viscosity of water (8.9 × 10^−4^ Pa∙s); Q is the volume of permeated pure water per unit of time (m^3^·s^−1^) and ∆P is the on-stream pressure (0.1 MPa).

#### 2.4.2. Filtering Performance Test

Penetration testing was performed using an ultrafiltration cup device (MSC050, Shanghai, China). All tests were performed at 0.1 MPa, and each sample membrane was pre-loaded with the deionized water for 20 min; filtration was carried out for 30 min and the filtrate volume was measured. Each trial was performed three times, and the mean value was obtained. The water flux in the ultrafiltration membrane was calculated according to Formula (3).

(3) $$J=\frac{V}{A\cdot t}$$

where J is the water flux (L·m^−2^·h^−1^); A is the membrane effective area (m^2^); V is the penetrating fluid volume (L) and t is the penetration time (h).

While evaluating the fouling resistance and retention properties, the above filtration experiments were carried out, and the stable membrane flux was recorded as J_1_. The feed solution was then replaced with a 1 g·L^−1^ BSA solution. Filtration was carried out for 90 min and the stable membrane flux was recorded as J_p_. The membrane was rinsed with deionized water and then the pure water flux was further checked. The final stable flux was recorded as J_2_. The rejection rate (R), the reversible flux recovery rate (FRR), the total fouling ratio (Rt), the irreversible fouling ratio (Rir) and the reversible fouling ratio (Rr) of the membrane were calculated using the following equations:

(4) $$R=\left(1-\frac{C_{p}}{C_{f}}\right)\times 100\%$$

where C_P_ and C_f_ represent the concentrations of the BSA infiltration solution and the original solution, respectively.

(5) $$\mathrm{FRR}=\frac{J_{2}}{J_{1}}\times 100\%$$

(6) $$\mathrm{Rt}=\frac{J_{1}-J_{p}}{J_{1}}\times 100\%$$

(7) $$\mathrm{Rir}=\frac{J_{1}-J_{2}}{J_{1}}\times 100\%$$

(8) $$\mathrm{Rr}=\frac{J_{2}-J_{p}}{J_{1}}\times 100\%$$

#### 2.4.3. Retention Accuracy Test

Protein solutions with four molecular weights were used to determine the retention accuracy of the different PVDF ultrafiltration membranes (bovine serum protein (BSA M_w_ = 68,000), ovalbumin (OVA M_w_ = 45,000), pepsin (PS M_w_ = 35,000) and trypsin (TPS M_w_ = 25,000)). A protein solution of 1.0 g L^−1^ was added to the ultrafiltration cup and prepressed for 30 min. Absorbance of the penetrating fluid was determined using a UV spectrophotometer, and C_p_ and C_f_ were calculated from the standard curve. Then, the rejection rate of the ultrafiltration membrane was calculated according to Formula (4).

#### 2.4.4. Antibacterial Test

The antibacterial activity of the different ultrafiltration membranes was measured by the disk diffusion method [20]. A model bacterial solution of 100 μL (10^6^ CFU·mL^−1^), containing either *Escherichia coli* or *Staphylococcus aureus*, was placed on a solidified agar plate and diffused under sterile conditions. After sterilization for 30 min, the PVDF ultrafiltration membrane, C_60_(OH)_n_/PVDF ultrafiltration membrane and C_60_(OH)_n_-Ag/PVDF ultrafiltration membrane with a diameter of 20 mm were inoculated on the inoculated agar plate at 37 °C for 24 h. The inhibition zone diameter of the different ultrafiltration membranes was measured on a millimeter scale.

## 3. Results and Discussion

**Figure 1:**
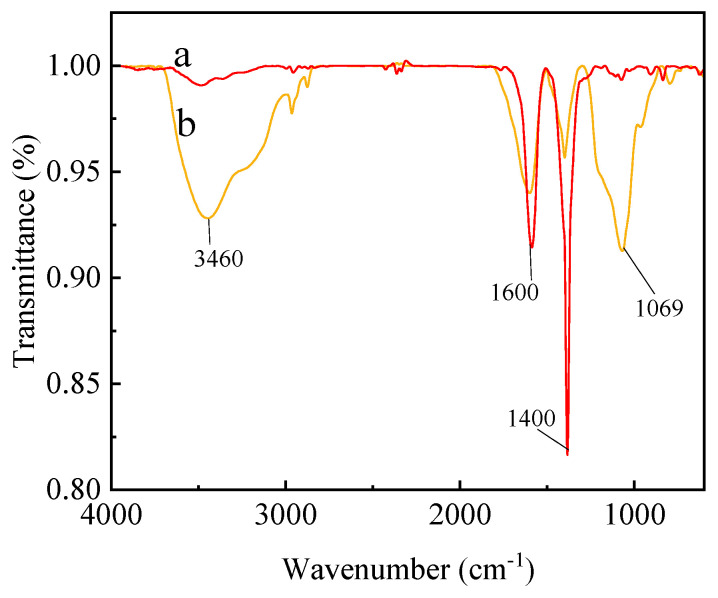
ATR-FTIR of C_60_(OH)_n_ (a) and the C_60_(OH)_n_-Ag complex (b).

The infrared spectra of C_60_(OH)_n_ and the C_60_(OH)_n_-Ag complex are shown in Figure 1. As shown in Figure 1a, an O-H absorption peak at 3460 cm^−1^, C=C stretching vibration peak at 1600 cm^−1^, C-O-H characteristic absorption peak at 1400 cm^−1^ and C-O characteristic absorption peak at 1069 cm^−1^ are the main characteristic peaks of C_60_(OH)_n_, which indicated that C_60_ was converted to C_60_(OH)_n_ [21]. In Figure 1b, the main absorption peak of the C_60_(OH)_n_-Ag complex did not shift in comparison with the characteristic absorption peak of C_60_(OH)_n_, indicating that C_60_(OH)_n_ existed in the C_60_(OH)_n_-Ag complex.

**Figure 2:**
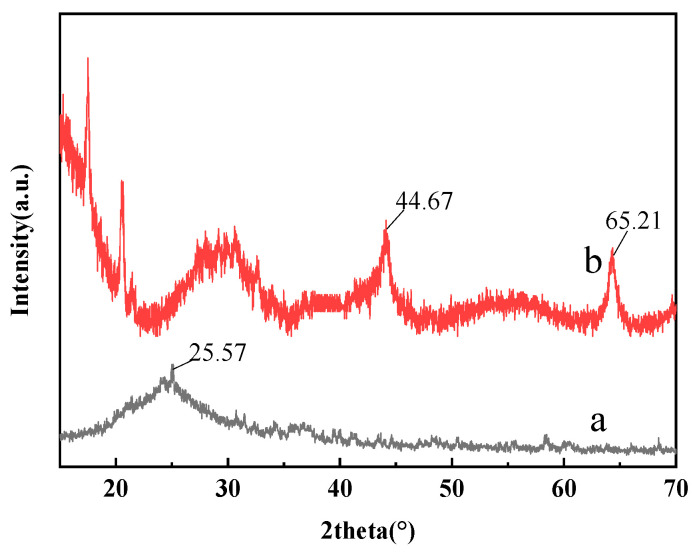
XRD of C_60_(OH)_n_ (a) C_60_(OH)_n_-Ag complex (b).

The XRD of the C_60_(OH)_n_ and the C_60_(OH)_n_-Ag complexes are shown in Figure 2. The characteristic peak of C_60_(OH)_n_ appears at 25.57 in Figure 2a [22], the characteristic peak of Ag occurs at 44.67° and 65.21° in Figure 2b [23]. After the formation of the C_60_(OH)_n_-Ag complex, a part of Ag entered the lattice of C_60_(OH)_n_ and replaced C, resulting in the deformation of the C_60_(OH)_n_ lattice. Therefore, the characteristic peak of the C_60_(OH)_n_-Ag complex was shifted (Figure 2b) [24], indicating that the C_60_(OH)_n_-Ag complex was synthesized.

**Figure 3:**
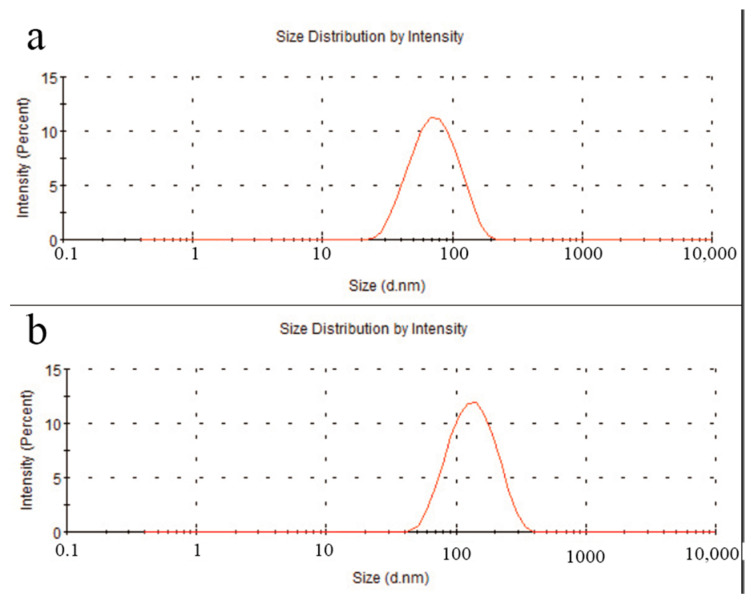
Particle size of C_60_(OH)_n_ (**a**) and the C_60_(OH)_n_-Ag complex (**b**).

After ultrasonic treatment in solution, the particle size of C_60_(OH)_n_ was around 78.1 nm (Figure 3a), while the particle size of the C_60_(OH)_n_-Ag complex was approximately 142 nm (Figure 3b). Both exhibited good dispersibility and uniform stability. Additionally, the increase in particle size indicated that Ag recombined successfully on the surface of C_60_(OH)_n_.

**Figure 4:**
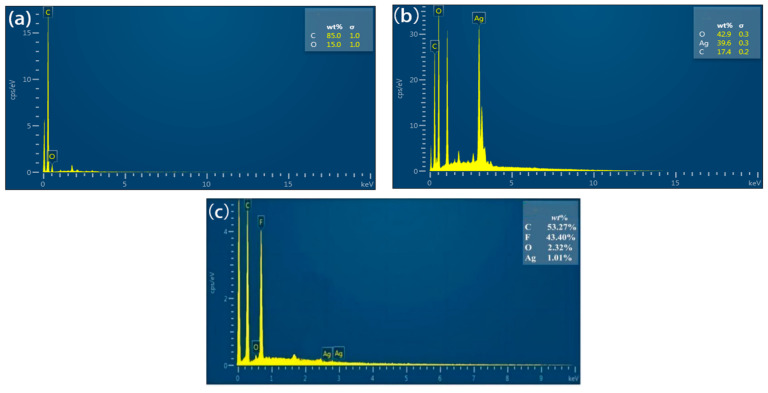
EDS of the modified C_60_(OH)_n_ (**a**), C_60_(OH)_n_-Ag (**b**) and C_60_(OH)_n_-Ag/PVDF ultrafiltration membranes (**c**).

Figure 4a,b shows that the C_60_(OH)_n_ contained C of 85 wt.% and O of 25 wt.%, while the C_60_(OH)_n_-Ag complex had O of 42.9 wt.%, Ag of 39.6 wt.% and C of 17.4 wt.%. The increase in O content in the C_60_(OH)_n_-Ag complex in comparison to C_60_(OH)_n_ was due to the presence of a small amount of water in the C_60_(OH)_n_-Ag complex. Figure 4c shows that the contents of the C, F, O and Ag elements in the C_60_(OH)_n_-Ag/PVDF ultrafiltration membrane were, respectively, 53.27 wt.%, 43.40 wt.%, 2.32 wt.% and 1.01 wt.%, indicating that the C_60_(OH)_n_-Ag complex was successfully incorporated into the PVDF ultrafiltration membrane.

**Figure 5:**
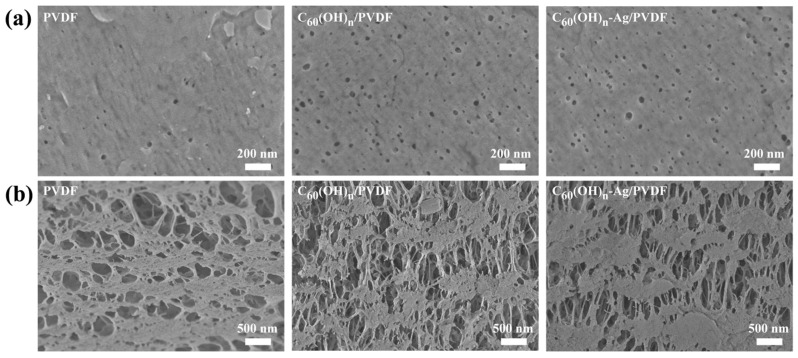
SEM images of the surface and cross-sectional morphology of different membranes: (**a**) surface and (**b**) cross-section.

**Figure 6:**
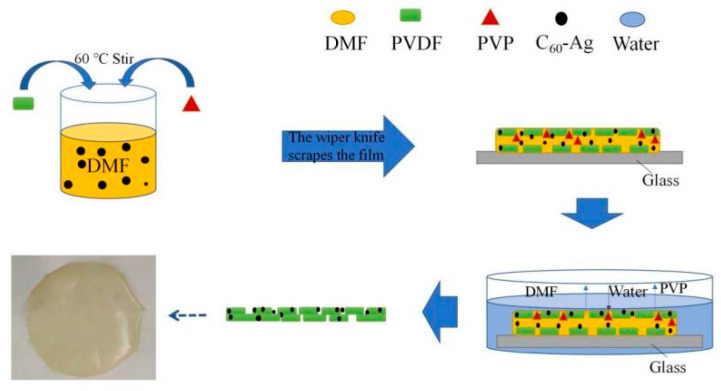
Film-forming mechanism of the C_60_(OH)_n_-Ag/PVDF ultrafiltration membrane.

The ultrafiltration membrane is composed of an epidermal layer and a supporting layer, and its surface or cross-sectional morphology has great influence on its properties. Figure 5 shows SEM images of the surface and cross-sectional morphology of the different ultrafiltration membranes. Compared with the PVDF ultrafiltration membrane, there were more micro pores in the surface layers of the C_60_(OH)_n_/PVDF ultrafiltration membrane and the C_60_(OH)_n_-Ag/PVDF ultrafiltration membrane, while the structures of their supporting layer changed from “spongy holes” to the coexistence state of “spongy holes” and “finger holes”, and many “finger holes” were added in the supporting layers. This is because the C_60_(OH)_n_ structure contained hydrophilic groups, such as hydroxyl groups, that could attract more water molecules during the film formation process, thus the solidification rate of the ultrafiltration membrane was sped up. In addition, with an increase in the number of oxygen-containing functional groups, the hydrophilic functional groups could form a water layer, with water molecules in the pores on the membrane surface and in the inner wall of the membrane, through van der Waals forces and hydrogen bonding, and the formation of the surface dense layer became denser, which significantly improved the hydrophilicity of the membrane. The formation process and structure simulation diagram of the C_60_(OH)_n_-Ag/PVDF ultrafiltration membrane are shown in Figure 6.

**Figure 7:**
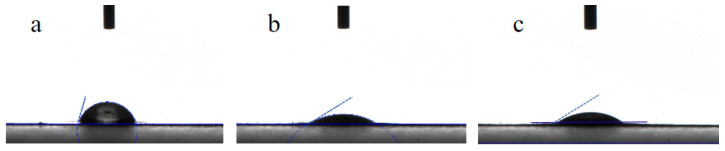
Contact angle of the different ultrafiltration membranes: (**a**) the PVDF ultrafiltration membrane, (**b**) the C_60_(OH)_n_/PVDF ultrafiltration membrane and (**c**) the C_60_(OH)_n_-Ag/ PVDF ultrafiltration membrane.

Contact angle is the most intuitive index of a substance’s hydrophilic properties: the smaller the contact angle, the better the hydrophilic properties [25]. As shown in Figure 7, the water contact angle of the PVDF ultrafiltration membrane was as high as 75.050°, which was due to the poor hydrophilicity of the PVDF polymer material. After the introduction of C_60_(OH)_n_ into PVDF, the water contact angle of the obtained C_60_(OH)_n_/PVDF ultrafiltration membrane was reduced to 33.173°. The water contact angle of the C_60_(OH)_n_-Ag/PVDF ultrafiltration membrane was 34.504°, which was like that of the C_60_(OH)_n_-modified PVDF ultrafiltration membrane, indicating that the great improvement in the hydrophilicity of the C_60_(OH)_n_-Ag/PVDF ultrafiltration membrane was related to C_60_(OH)_n_ and had little relationship with the introduction of silver.

Mechanical properties are important parameters to evaluate the applicability of an ultrafiltration membrane: the stronger the tensile resistance, the longer its service life. It can be seen from Table 1 that the tensile strength and elongation at break of the C_60_(OH)_n_/PVDF and C_60_(OH)_n_-Ag/PVDF ultrafiltration membranes were reduced in comparison with the PVDF ultrafiltration membrane, which could be attributed to the complex mechanical properties of nanocomposite membranes [16]. First, the polymer/nanoparticulate interface characteristics could affect the mechanical properties of the nanocomposite membranes. Accordingly, the incompatibility between the nanoparticles and the PVDF matrix would result in a failure in external force conversion from the PVDF matrix to the inorganic nanoparticles. Second, when the nanoparticles were loaded onto the PVDF ultrafiltration membrane, the agglomeration of nanoparticles would lead to poor compatibility in the polymer matrix, which would reduce the modulus of the PVDF ultrafiltration membrane [26].

**Figure 8:**
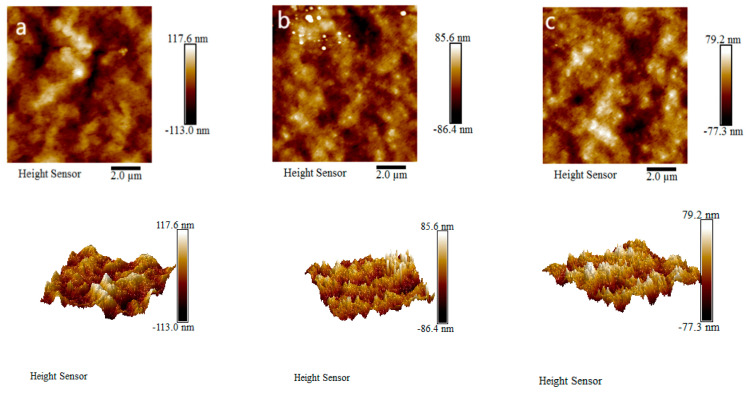
Two-dimensional and three-dimensional AFM graphs of the different ultrafiltration membranes: (**a**) the PVDF ultrafiltration membrane, (**b**) the C_60_(OH)_n_/PVDF ultrafiltration membrane and (**c**) the C_60_(OH)_n_-Ag/PVDF ultrafiltration membrane.

Roughness of the membrane surface is a key factor used to determine the anti-pollution performance of ultrafiltration membranes. If the roughness of the membrane surface is greater, more organic pollutants will be likely to accumulate in the depressions of the membrane surface [27,28,29]. AFM images of the PVDF ultrafiltration membrane, C_60_(OH)_n_/PVDF ultrafiltration membrane and C_60_(OH)n-Ag/PVDF ultrafiltration membrane are shown in Figure 8, and the detailed calculation results of the surface roughness parameters of the different ultrafiltration membranes are shown in Table 2. It can be seen from Figure 8 that the three ultrafiltration membranes all had characteristic peak–valley structures. Surface roughness values of the C_60_(OH)_n_/PVDF ultrafiltration membrane and the C_60_(OH)_n_-Ag/PVDF ultrafiltration membrane were, respectively, reduced by 8.1 nm and 10.1 nm in comparison with the PVDF ultrafiltration membrane, as shown in Table 2; the reductions in surface roughness in the PVDF ultrafiltration membrane modified by C_60_(OH)_n_ or C_60_(OH)_n_-Ag were due to the movement of part of C_60_(OH)_n_ or C_60_(OH)_n_-Ag to the surface of the ultrafiltration membrane during the phase conversion process.

**Figure 9:**
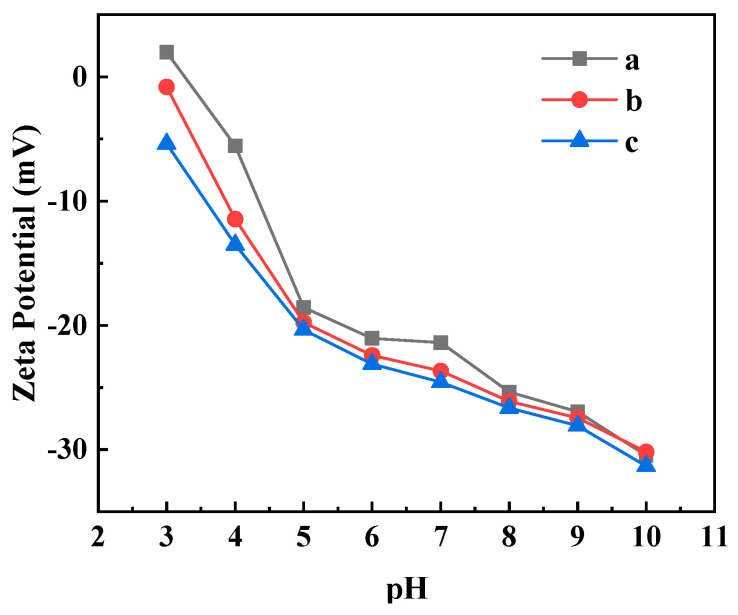
Relationship between Zeta potential and pH change in the ultrafiltration membranes: (a) the PVDF ultrafiltration membrane, (b) the C_60_(OH)_n_/PVDA ultrafiltration membrane and (c) the C_60_(OH)_n_-Ag/PVDF ultrafiltration membrane.

Zeta potential is measured by the adsorption of ions in the solution by the charging action, and the potential difference between the membrane surface and the solution can reflect the anti-pollution ability of the ultrafiltration membrane [30,31]. The relationship between the Zeta potential and pH of the different ultrafiltration membranes are shown in Figure 9. From Figure 9, the Zeta potential of the PVDF ultrafiltration membrane was positive when pH = 3 and negative when pH = 4–10. The Zeta potentials of the C_60_(OH)_n_/PVDF ultrafiltration membrane and the C_60_(OH)_n_-Ag/PVDF ultrafiltration membrane were negative when pH = 3–10. The Zeta potential of the three ultrafiltration membranes decreased with an increase in pH, and the charge density order of the membrane surface was: C_60_(OH)_n_-Ag/PVDF ultrafiltration membrane > C_60_(OH)_n_/PVDF ultrafiltration membrane > PVDF ultrafiltration membrane. Electrostatic repulsion easily occurred between the negatively charged ultrafiltration membrane and negatively charged macromolecular proteins. The higher the surface charge density of the membrane, the stronger the electrostatic repulsion effect of the ultrafiltration membrane on the protein pollutants [32]. In addition, the increase in charge density also influenced the early adhesion and colonization of bacteria, which could hinder the formation of biofilms and reduce the adhesion of bacteria to objects.

**Figure 10:**
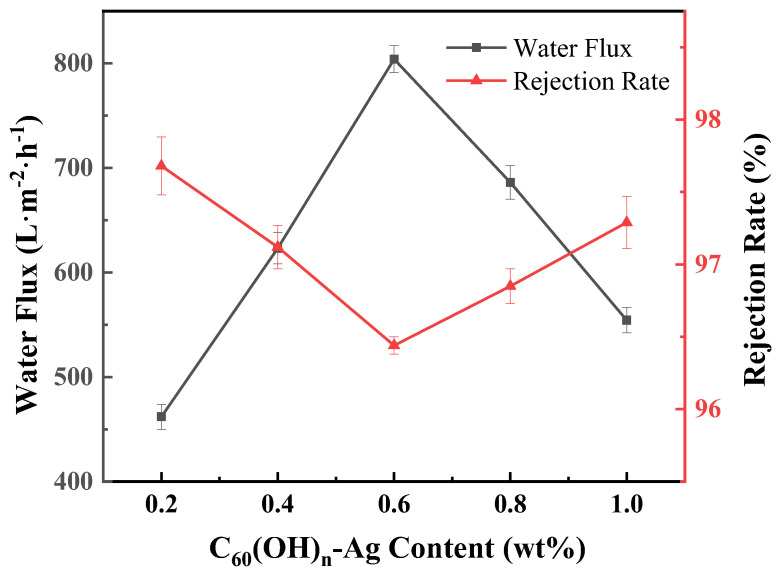
Effect of C_60_(OH)_n_-Ag content on water flux and rejection in the C_60_(OH)_n_-Ag/PVDF ultrafiltration membrane.

Pure water flux and retention rate are the main indexes used to evaluate a membrane’s filtration performance. In Table 3, compared with the PVDF ultrafiltration membrane, the water flux of the C_60_(OH)_n_/PVDF ultrafiltration membrane and the C_60_(OH)_n_-Ag/PVDF ultrafiltration membrane increased to more than three times; their retention rates were increased by 17.52% and 21.44%, respectively, and the filtration performance of the ultrafiltration membrane was greatly improved. The increases in pure water flux in the C_60_(OH)_n_/PVDF ultrafiltration membrane and the C_60_(OH)_n_-Ag/PVDF ultrafiltration membrane were due to increases in their average porosities and hydrophilicities; in addition, this could also be attributed to the dense hydration layer formed by hydrogen bond interactions between C_60_(OH)_n_ nanoparticles and water molecules. Figure 10 shows that, with an increase in C_60_(OH)_n_-Ag content, the water flux of the C_60_(OH)_n_-Ag/PVDF ultrafiltration membrane first increased and then decreased, while the retention rate was the opposite. When the content of C_60_(OH)_n_-Ag was 0.6 wt.%, the lowest retention rate of the C_60_(OH)_n_-Ag/PVDF ultrafiltration membrane was 96.44%, which fully met the requirement for ultrafiltration membrane retention rates.

In recent years, the use of inorganic carbon nanoparticles to modify PVDF ultrafiltration membranes has attracted the attention of researchers in the same field. The prepared C_60_(OH)_n_-Ag/PVDF ultrafiltration membrane in this study had a lower contact angle than the other membranes, and the water flux was higher than the others when the rejection rate of BSA was maintained above 90%. These results are shown in Table 4.

The evaluation of the separation accuracy of an ultrafiltration membrane is based on a retention rate of more than 90% of macromolecular substances. Retention rates of the PVDF ultrafiltration membrane, C_60_(OH)_n_/PVDF ultrafiltration membrane and C_60_(OH)_n_-Ag/PVDF ultrafiltration membrane with BSA, OVA, PS and TPS are shown in Table 5. It can be seen from Table 5 that the C_60_(OH)_n_-Ag/PVDF ultrafiltration membrane had the best interception effects on BSA, OVA, PS and TPS, while the C_60_(OH)_n_/PVDF ultrafiltration membrane was superior to the PVDF ultrafiltration membrane. Retention rates of the PVDF and C_60_(OH)_n_/PVDF ultrafiltration membranes on OVA, PS and TPS were lower than 90%, which did not meet the separation requirements. The rejection rate of the C_60_(OH)_n_/PVDF ultrafiltration membrane with BSA was more than 90%, and the separation accuracy was 68,000 Da. The interception rates of the C_60_(OH)_n_-Ag/PVDF ultrafiltration membrane with OVA and PS were higher than 90%, the membrane’s interception rate with TPS was lower than 90% and the separation accuracy of the C_60_(OH)_n_-Ag/PVDF ultrafiltration membrane could reach 35,000 Da.

**Figure 11:**
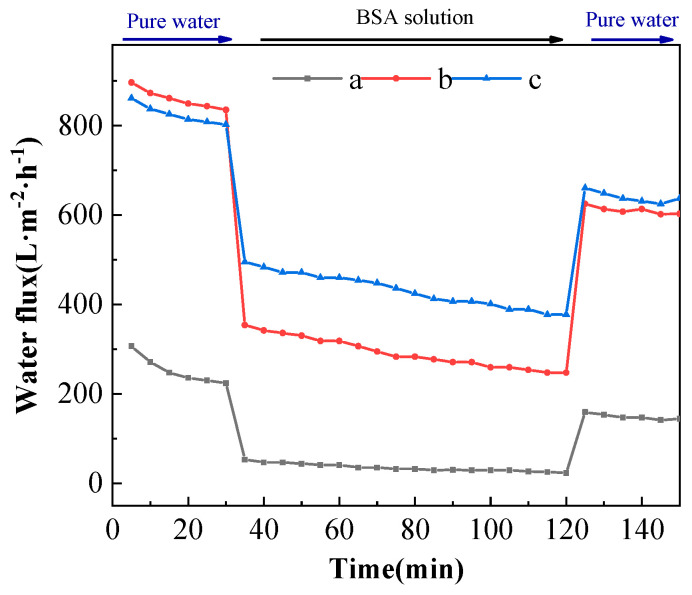
Permeation flux of different ultrafiltration membranes as a function of time in the BSA cycling assay. (a) The PVDF ultrafiltration membrane, (b) the C_60_(OH)_n_/PVDA ultrafiltration membrane and (c) the C_60_(OH)_n_-Ag/PVDF ultrafiltration membrane.

The changes in the permeability flux of different ultrafiltration membranes with time spent in the BSA solution cycling test are shown in Figure 11. From Figure 11, the permeation fluxes of the PVDF ultrafiltration membrane, C_60_(OH)_n_-Ag/PVDF ultrafiltration membrane and C_60_(OH)_n_-Ag/PVDF ultrafiltration membrane showed a trend of first decreasing and then stabilizing during the 90-minute filtration of the BSA solution. The permeation fluxes of the three ultrafiltration membranes were, respectively, stable at around 25.36 L·m^−2^·h^−1^, 247.70 L·m^−2^·h^−1^ and 377.45 L·m^−2^·h^−1^. Compared with the PVDF ultrafiltration membrane, the C_60_(OH)_n_-Ag/PVDF ultrafiltration membrane and the C_60_(OH)_n_-Ag/PVDF ultrafiltration membrane both had larger permeation fluxes after filtration for 90 min with the BSA solution. This is because the addition of C_60_(OH)_n_ and C_60_(OH)_n_-Ag could improve the hydrophilicity of the membrane surface and promote the formation of a hydration layer on the membrane surface; thus, it was difficult for BSA molecules to enter the membrane pore and block the membrane pore. On the other hand, more finger-like pore structures were formed in the supporting layer, which could provide a greater permeability flux. In addition, the pure water flux of each ultrafiltration membrane after washing was not completely restored to the initial flux, which was due to the inevitable adhesion and adsorption of protein molecules onto the membrane surface and into the membrane pores.

**Figure 12:**
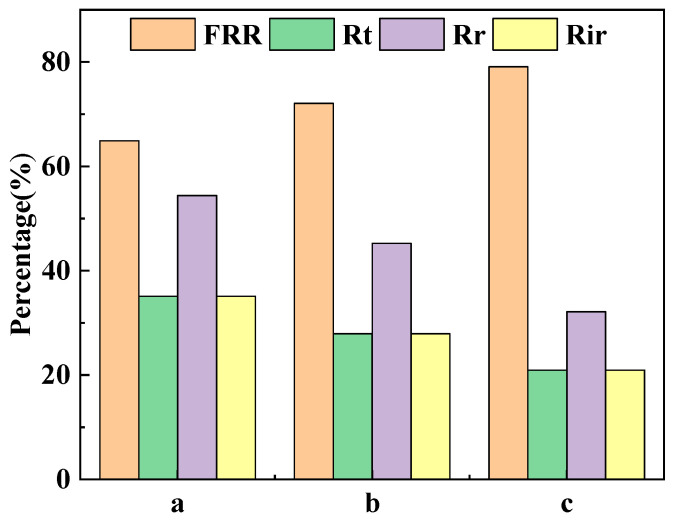
Flux recovery rate (FRR), proportion of total fouling (Rt), proportion of irreversible fouling (Rir) and proportion of reversible fouling (Rr) in the different ultrafiltration membranes after cyclic filtration. (a) The PVDF ultrafiltration membrane, (b) the C_60_(OH)_n_/PVDA ultrafiltration membrane and (c) the C_60_(OH)_n_-Ag/PVDF ultrafiltration membrane.

Membrane fouling refers to the deposition of pollutants on the surface of the membrane or inside the membrane pores, resulting in a temporary or permanent deterioration of the membrane flux. Membrane contamination is divided into reversible contamination, which can be recovered by operations such as washing, and irreversible contamination, which causes a permanent loss of membrane properties. The proportions of FRR, Rt, Rir and Rr of the different ultrafiltration membranes after cyclic filtration are shown in Figure 12. The FRRs of the PVDF ultrafiltration membrane, C_60_(OH)_n_/PVDF ultrafiltration membrane and C_60_(OH)_n_-Ag/PVDF ultrafiltration membrane were, respectively, 64.91%, 74.46% and 79.08%, and their Rir values were, respectively, 35.08%, 27.93% and 20.91%. The increase in FRR and the decrease in Rir values indicated that the anti-pollution abilities of the C_60_(OH)_n_/PVDF ultrafiltration membrane and the C_60_(OH)_n_-Ag/PVDF ultrafiltration membrane were improved. On the one hand, the addition of C_60_(OH)_n_ or the C_60_(OH)_n_-Ag complex in the PVDF ultrafiltration membrane improved the hydrophilicity of the membrane surface and its ability to form a hydration layer. On the other hand, the surface roughness of the membrane became smaller (Table 2), which reduced the attachment of pollutants. In addition, the pH of the 1 g·L^−1^ of aqueous BSA solution was about 7; both the C_60_(OH)_n_/PVDF ultrafiltration membrane and the C_60_(OH)_n_-Ag/PVDF ultrafiltration membrane had strong surface charge densities under this condition (Figure 9), which had a stronger electrostatic repulsion to negatively charged BSA molecules.

**Figure 13:**
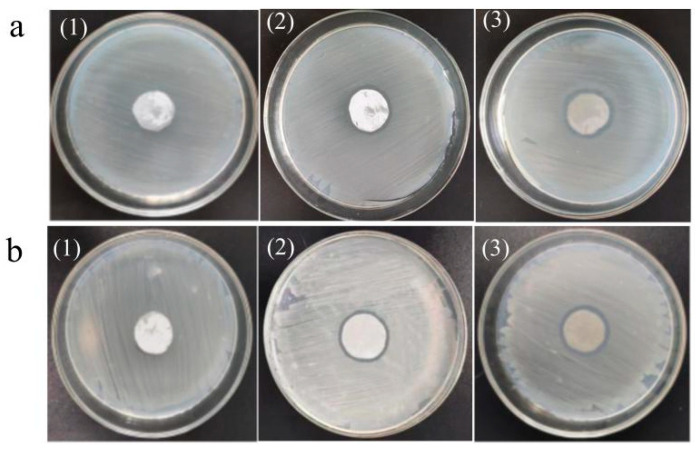
Bacteriostatic effects of the different ultrafiltration membranes on *Escherichia coli* (**a**) and *Staphylococcus aureus* (**b**). (1) The PVDF ultrafiltration membrane, (2) the C_60_(OH)_n_/PVDF ultrafiltration membrane, and (3) the C_60_(OH)_n_-Ag/PVDF ultrafiltration membrane.

Figure 13 shows the bacteriostatic effects of the PVDF ultrafiltration membrane, C_60_(OH)_n_/PVDF ultrafiltration membrane and C_60_(OH)_n_-Ag/PVDF ultrafiltration membrane on *Escherichia coli* and *Staphylococcus aureus*, and the diameter of their bacteriostatic circles are shown in Table 6. From Table 6, the diameters of the inhibition zones on the PVDF membrane, C_60_(OH)_n_/PVDF membrane and C_60_(OH)_n_-Ag/PVDF membrane against *E. coli* were, respectively, 20 ± 0.00 mm, 21 ± 0.51 mm and 23 ± 0.35 mm; the diameters of the inhibition zones against *Staphylococcus aureus* were, respectively, 20 ± 0.00 mm, 21 ± 0.43 mm and 23 ± 0.45 mm. It can be seen that the PVDF ultrafiltration membrane had no inhibitory effect on the two bacteria species, while the C_60_(OH)_n_/PVDF ultrafiltration membrane and the C_60_(OH)_n_-Ag/PVDF ultrafiltration membrane had a certain inhibitory effect on the two bacteria, and the antibacterial effect of the C_60_(OH)_n_-Ag/PVDF ultrafiltration membrane was significantly higher than that of the C_60_(OH)_n_/PVDF ultrafiltration membrane. Compared with the C_60_(OH)_n_/PVDF ultrafiltration membrane, the antibacterial properties of the C_60_(OH)_n_-Ag/PVDF ultrafiltration membrane were derived from the dual effect of C_60_(OH)_n_ and Ag. Some studies have shown that C_60_ can activate oxygen and produce superoxide anion radicals by generating singlet oxygen molecules, which are involved in the inactivation of bacteria [16]. Silver has an excellent antibacterial effect, and it can kill bacteria by being in contact with microbial cells and destroying their cellular structure.

## 4. Conclusions

The C_60_(OH)_n_-Ag complex prepared in this study had good dispersion and homogeneous stability, demonstrating its excellence as a possible modifier. The C_60_(OH)_n_-Ag/PVDF ultrafiltration membrane was prepared by blending C_60_(OH)_n_-Ag complexes with PVDF. The obtained C_60_(OH)_n_-Ag/PVDF ultrafiltration membrane had improved hydrophilicity, a smooth surface morphology and finger-like pore structures. The pure water flux of the C_60_(OH)_n_-Ag/PVDF ultrafiltration membrane was significantly higher than that of the PVDF ultrafiltration membrane (804.05 L·m^−2^·h^−1^), and the BSA retention rate remained above 90%. The addition of C_60_(OH)_n_-Ag had a better effect on the fouling resistance of the C_60_(OH)_n_-Ag/PVDF ultrafiltration membrane. The FRR of the C_60_(OH)_n_-Ag/PVDF ultrafiltration membrane was higher than that of the PVDF ultrafiltration membrane. The C_60_(OH)_n_-Ag/PVDF ultrafiltration membrane had a good inhibition effect on *Escherichia coli* and *Staphylococcus aureus*. In further studies, we will continue to broaden the application of this new membrane in different types of wastewater, which will be an interesting topic.

## Figures and Tables

**Table 1:** The tensile strength and elongation at break of the different ultrafiltration membranes.

Samples	Cross-Sectional Area/mm^2^	Tensile Strength/N·mm^−2^	Elongation at Break/%
PVDF ultrafiltration membrane	1.00	6.00	21.50
C_60_(OH)_n_/PVDF ultrafiltration membrane	1.00	4.82	17.05
C_60_(OH)_n_-Ag/PVDF ultrafiltration membrane	1.00	5.18	19.02

**Table 2:** Surface average roughness (Ra) for the three kinds of ultrafiltration membranes.

Samples	Ra (nm)
PVDF ultrafiltration membrane	34.5
C_60_(OH)_n_/PVDF ultrafiltration membrane	26.4
C_60_(OH)_n_-Ag/PVDF ultrafiltration membrane	24.4

**Table 3:** Parameters related to the ultrafiltration membrane.

Samples	Mean Porosity (ε (%))	Mean Aperture (r (nm))	Water Flux (L·m^−2^·h^−1^)	Rejection Rate (%)
PVDF	68.50	54.91	224.11	75.00
C_60_(OH)_n_/PVDF	79.53	40.52	835.50	92.52
C_60_(OH)_n_-Ag/PVDF	75.88	39.22	804.05	96.44

**Table 4:** Different performance parameters of modified PVDF membranes reported in the literature.

Membrane	Water Contact Angle (°)	Water Flux (L·m^−2^·h^−1^)	BSA Rejection (%)	Ref.
PSBMA@GO/PVDF	64.7°	50	96	[7]
PVDF-SGO	50°	740	98	[8]
PVDF/GONRs/PVP	56.5°	532.28	98	[9]
QGO/PVDF	55.4°	981~1285	84	[6]
MWCNTs-COOH/PVDF	74°	250–350	Close to 100	[10]
PMWCNT/PVDF	66.6°	384	91	[11]
PVDF/MWCNT/Polypyrrole	65°	554.3	----	[33]
PVDF/GO-CuO	60.53°	178.8	94.28	[34]
TiO_2_-HNTs/PVDF	59.7°	354.2	75	[35]
TiO2-HAP@PVDF	74°	350	58.75	[36]
C_60_(OH)_n_-Ag/PVDF	34.5°	804.05	96.44	This work

**Table 5:** Retention rate of the different ultrafiltration membranes on different proteins.

	Samples	PVDF Ultrafiltration Membrane	C_60_(OH)_n_/PVDF Ultrafiltration Membrane	C_60_(OH)_n_-Ag/PVDF Ultrafiltration Membrane
Rejection Rate				
BSA %		75.00	92.52	96.44
OVA %		65.60	88.80	92.23
PS %		64.84	86.61	90.36
TPS %		35.81	52.41	57.44

**Table 6:** Antimicrobial diameter of the different ultrafiltration membranes against *Escherichia coli* and *Staphylococcus aureus*.

Samples	Antimicrobial Diameter D/mm	
	*Escherichia coli*	*Staphylococcus aureus*
PVDF ultrafiltration membrane	20 ± 0.00	20 ± 0.00
C_60_(OH)_n_/PVDF ultrafiltration membrane	21 ± 0.51	21 ± 0.43
C_60_(OH)_n_-Ag/PVDF ultrafiltration membrane	23 ± 0.35	23 ± 0.45

## Data Availability

The original contributions presented in this study are included in the article. Further inquiries can be directed to the corresponding author.
